# Chloride as a Beneficial Macronutrient in Higher Plants: New Roles and Regulation

**DOI:** 10.3390/ijms20194686

**Published:** 2019-09-21

**Authors:** José M. Colmenero-Flores, Juan D. Franco-Navarro, Paloma Cubero-Font, Procopio Peinado-Torrubia, Miguel A. Rosales

**Affiliations:** 1 Instituto de Recursos Naturales y Agrobiología, Spanish National Research Council (CSIC), Avda Reina Mercedes 10, 41012 Sevilla, Spain; juande@irnase.csic.es (J.D.F.-N.); paloma.cubero-font@supagro.fr (P.C.-F.); ppeinado@irnas.csic.es (P.P.-T.); mrosales@irnas.csic.es (M.A.R.); 2 Biochimie et physiologie Moléculaire des Plantes (BPMP), Univ Montpellier, CNRS, INRA, SupAgro, 2 place P. Viala, 34060 Montpellier, France

**Keywords:** chloride homeostasis, beneficial macronutrient, ion transport, anion channels, plant nutrition, turgor, nitrate, water balance, WUE, NUE, stress

## Abstract

Chloride (Cl^−^) has traditionally been considered a micronutrient largely excluded by plants due to its ubiquity and abundance in nature, its antagonism with nitrate (NO_3_^−^), and its toxicity when accumulated at high concentrations. In recent years, there has been a paradigm shift in this regard since Cl^−^ has gone from being considered a harmful ion, accidentally absorbed through NO_3_^−^ transporters, to being considered a beneficial macronutrient whose transport is finely regulated by plants. As a beneficial macronutrient, Cl^−^ determines increased fresh and dry biomass, greater leaf expansion, increased elongation of leaf and root cells, improved water relations, higher mesophyll diffusion to CO_2_, and better water- and nitrogen-use efficiency. While optimal growth of plants requires the synchronic supply of both Cl^−^ and NO_3_^−^ molecules, the NO_3_^−^/Cl^−^ plant selectivity varies between species and varieties, and in the same plant it can be modified by environmental cues such as water deficit or salinity. Recently, new genes encoding transporters mediating Cl^−^ influx (*ZmNPF6.4* and *ZmNPF6.6*), Cl^−^ efflux (*AtSLAH3* and *AtSLAH1*), and Cl^−^ compartmentalization (*AtDTX33*, *AtDTX35*, *AtALMT4,* and *GsCLC2*) have been identified and characterized. These transporters have proven to be highly relevant for nutrition, long-distance transport and compartmentalization of Cl^−^, as well as for cell turgor regulation and stress tolerance in plants.

## 1. Introduction

The chloride (Cl^−^) anion is the dominant form of the halogen element chlorine in soils. Especially in the agronomic context, Cl^−^ has traditionally been considered a toxic anion rather than a plant nutrient. This is a consequence of two main reasons: Toxicity resulting from excessive Cl^−^ accumulation in sensitive organs under salt stress conditions, and the widespread belief that Cl^−^ and nitrate (NO_3_^−^) are antagonistic molecules. As a result, root Cl^−^ uptake and accumulation occurs to the detriment of nitrate (NO_3_^−^) nutrition, an important source of nitrogen (N) for higher plants. However, unlike NO_3_^−^, Cl^−^ is one of the 16 essential elements for plant growth. Since Cl^−^ is supposedly needed only in small quantities for healthy growth of plants (about 50–100 μM in the nutrient media), it is classified as a micronutrient [1]. Consequently, the vast majority of published information on the role and effects of Cl^−^ in higher plants deals with two extreme situations: Its function as an essential micronutrient and its toxicity under salt stress conditions.

Recently, Cl^−^ has been described as a beneficial element for the adequate development of plants when it is accumulated to macronutrient levels [2,3,4]. While many agronomic studies have also reported a substantial increase in yield for many crops in response to Cl^−^ fertilization [5], it was unclear the physiological processes affected, or to what extent the beneficial effects are Cl^−^-specific or associated with the accompanying cations. It is therefore necessary to expand our knowledge on: (i) The identification of the biological functions requiring macronutrient Cl^−^ levels; (ii) the degree of Cl^−^ specificity in these processes; (iii) the identification of genes encoding Cl^−^ membrane transporters relevant for plant nutrition; (iv) the signal-transduction pathways regulating Cl^−^ nutrition processes; and (v) a better understanding of the Cl^−^ versus NO_3_^−^ in vivo interaction. The fact that *Arabidopsis thaliana* is a poor Cl^−^ includer (Table 1; Section 4.2) may have hampered the identification of genes specifically involved in Cl^−^ nutrition mechanisms. The availability of the genome sequence of many other plant species and varieties, including crops, with contrasting Cl^−^ inclusion abilities is expected to rapidly allow the identification of new genes and alleles involved in the regulation of Cl^−^ homeostasis in higher plants.

These new focuses of interest, including the identification of new genes involved in Cl^−^ transport, have revived the interest of physiologists and molecular biologists in this essential nutrient. Excellent reviews have been published regarding the origin and abundance of chlorine in the environment, its function as a mineral micronutrient for plants, the occurrence and effects of Cl^−^ deficiency, its distribution in the plant, its toxicity under saline stress conditions, and the identification of genes involved in Cl^−^ exclusion mechanisms [3,4,5,6,7,8,9,10,11,12]. In this review we will particularly cover: The new vision of Cl^−^ as a beneficial macronutrient, away from the classical view as a toxic element for agriculture; the proposed biological functions in which Cl^−^ is involved as a beneficial macronutrient; the gene families implicated in the regulation of Cl^−^ transport, mainly in light of the recently identified genes; and their role in nutritional, biochemical, and stress-acclimatization functions.

## 2. Cl^−^ as an Essential Micronutrient

The Cl^−^ ion is an essential cofactor for oxygen evolution of photosystem II (PSII) in the chloroplast, stabilizing the water splitting system at the oxidizing site of PSII. Two Cl^−^ molecules are required to maintain the coordination structure of the Mn(4)Ca cluster [13], facilitating the proton flux from the water oxidation complex to the thylakoid lumen, thereby keeping the oxygen-evolving complex fully active (reviewed in [3]). Chloride also regulates the activity of some enzymes such as the asparagine synthetase [14], and the vacuolar proton-pumping ATPase [15]. A role of Cl^−^ in regulating amylase activity has also been proposed [16]. To ensure these cellular functions, only micromolar amounts of Cl^−^ are required in glycophyte plants. Accordingly, it has been generally accepted that the minimum Cl^−^ requirement for adequate plant growth in most plant species is in the range of 0.2–0.4 mg·g^−1^ dry weight (mg·g^−1^ DW) [1,12,17], which corresponds to the content of a micronutrient. Chloride is sufficiently abundant in nature to fulfil these requirements [7].

## 3. Cl^−^ as a Beneficial Macronutrient

**Figure 1:**
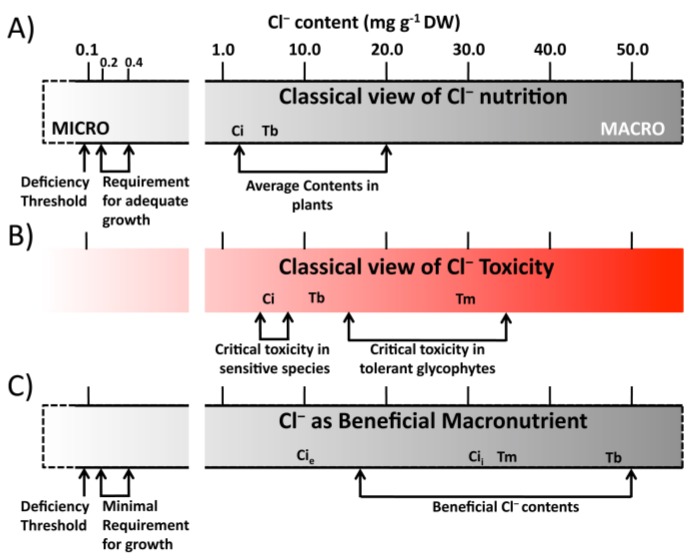
Cl^−^ homeostasis in glycophyte plants. Schematic illustration of Cl^−^ homeostasis in glycophyte plants according to the classical view of Cl^−^ nutrition (**A**), the classical view of Cl^−^ toxicity (**B**), and comparison with the recently proposed role of Cl^−^ as a beneficial macronutrient in glycophyte plants (**C**). (**A**,**B**) have been made from the data of Cl^−^ contents reported in different plant species, obtained from the review by Xu et al. [5]. Cl^−^ homeostasis as a beneficial macronutrient in glycophyte plants is illustrated in (**C**), according to the data recently reported [2,18,19,20]. Ci, citrus; Tb, tobacco; Tm, tomato; Ci_e_, citrus excluder genotypes; Ci_i_, citrus includer genotypes.

Despite the supposed low requirements, average Cl^−^ content in plants is much higher than the concentration required as a micronutrient [5]. It is actually the most abundant inorganic anion in plant cells when this nutrient is available at concentrations present in most environments [12]. Surprisingly, these Cl^−^ contents overlap with those reported as toxic to many plant species [5,7,12]. While average Cl^−^ content in plants varies in the range of 2.0–20.0 mg·g^−1^ DW (Figure 1A), the critical tissue Cl^−^ content for toxicity previously reported is about 4–7 and 15–35 mg·g^−1^ DW for Cl^−^-sensitive and Cl^−^-tolerant glycophyte species, respectively (Figure 1B). Thus, according to this traditional vision of plant Cl^−^ homeostasis, adequate plant development requires micronutrient Cl^−^ contents. However, plants accumulate about 10 to 100 times higher concentrations despite being toxic to many species. This vision implies that plants are unable to adequately regulate optimal levels of Cl^−^ and, as a consequence, the dominant homeostatic strategy should be the exclusion of this element. This is, in fact, the common view that currently exists about Cl^−^ management in agriculture.

Recent reports have shown that prolonged treatments with a nutrient solution containing Cl^–^ in the low milli-molar range (e.g., 4–5 mM Cl^−^) determine leaf Cl^−^ accumulation values between 25 and 50 mg·g^−1^ DW in different plant species (Figure 1C; Table 1). Despite these Cl^–^ contents clearly exceed the critical toxicity values mentioned above, these plants develop normally and grow without apparent symptoms of stress [2,18,19,20]. Root Cl^−^ uptake and long-distance transport require a considerable use of metabolic energy [18,21,22], clearly indicating that shoot Cl^−^ accumulation to macronutrient levels responds to specific biological adaptations. Under these conditions, Cl^−^ plays specific physiological roles that result in increased dry biomass and improved plant performance [2,23]. Beneficial elements are defined as those elements that stimulate growth, but are not essential in certain plant species, or under specific conditions [24]. Since Cl^−^ is not an essential macronutrient but it stimulates growth when accumulated to macronutrient levels, in addition to an essential micronutrient, Cl^−^ has been defined as a beneficial macronutrient [2].

**Figure 2:**
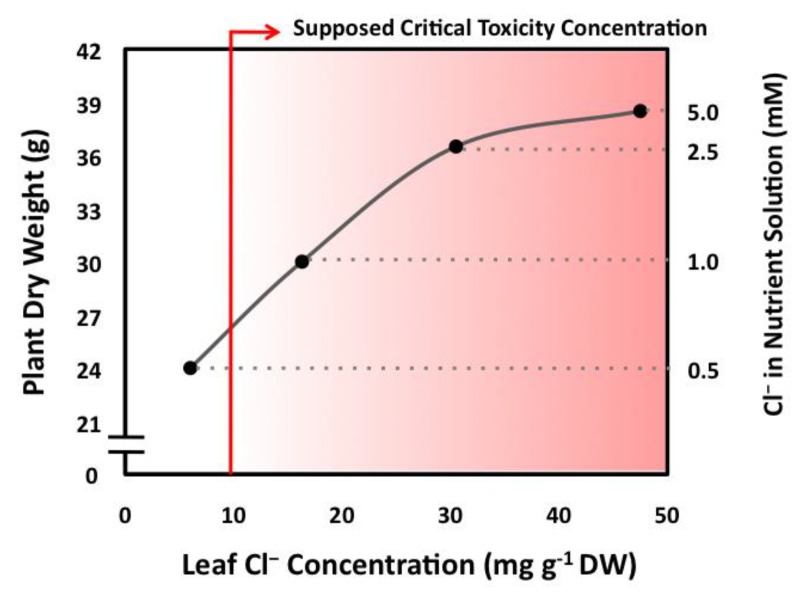
Stimulation of tobacco plant growth by macronutrient Cl^−^ contents. The data, obtained from Franco-Navarro et al. [2] and from Colmenero-Flores et al (umpublished results) illustrates growth stimulation in terms of dry weight biomass in a range of leaf Cl^−^ contents within the beneficial macronutrient range, which clearly overlaps with the previously reported toxic contents for this species [5]. There is a widespread belief that the accumulation of Cl^−^ in plant tissues does not necessary reflect the quantity requested. Chloride applications in the 1–5 mM Cl^−^ range determine increasing values of leaf Cl^−^ accumulation in a linear fashion, which in turn determine positive responses in terms of dry biomass, strongly supporting that plants regulate the required amount of Cl^−^ within the beneficial macronutrient range. Although the response remains positive with the application of 5 mM Cl^−^, the response curve inflection indicates that the trend can be reversed at higher Cl^−^ concentrations. For example, in a similar assay, in comparison to the 5 mM Cl^−^ treatment, 15 mM Cl^−^ application resulted in lower dry weight, indicating that this concentration exceeds the beneficial range of Cl^−^ nutrition.

The growth of tobacco plants is stimulated by Cl^−^ contents up to values of around 50 mg·g^−1^ DW (Figure 2; [2]), which is 5-fold the critical toxicity threshold previously reported for this species (Figure 1B; [5]). This apparent discrepancy may be due to the type of treatment applied. While prolonged treatments below 5–10 mM Cl^−^ can determine high leaf accumulations with no stress symptoms and/or positive growth responses, shorter salt stress treatments above 10–15 mM Cl^−^ can produce symptoms of toxicity with relatively low leaf Cl^−^ contents [5,25,26,27,28,29]. This is indicative that moderate Cl^−^ applications enable adequate transport and distribution of Cl^−^ at the subcellular, organ, and whole-plant levels (e.g., optimal rates of root uptake, xylem translocation, shoot accumulation, and intracellular compartmentalization). For example, after 30 weeks of undergoing a nutritional treatment of 4.5 mM Cl^−^, different varieties of citrus, a supposedly Cl^−^-sensitive crop, accumulated between 150 and 425 mM Cl^−^ in their leaf tissues with no symptoms of salt stress [18].

In summary, at concentrations commonly present in soils, in excess to those needed to satisfy micronutrient requirements but insufficient to cause toxicity (e.g., in a beneficial range of around 1–5 mM; [2]), plants accumulate Cl^−^ to macronutrient levels, leading to a better plant performance. Known or proposed biological processes favored by macronutrient Cl^−^ levels are explained below.

### 3.1. Charge–Balance, Osmoregulation, Turgor, Cell Volume, and Growth

Besides the micronutrient functions previously described, other roles largely attributed to Cl^−^ are the regulation of cell osmolarity and the electrical charge balance of cations [6,12]. Being a non-assimilating highly mobile anion, Cl^−^ is the preferred molecule to balance the electric charges of important cations such as potassium (K^+^), calcium (Ca^2+^), and protons (H^+^), playing important roles in the stabilization of the electric potential of cell membranes and the regulation of pH gradients and electrical excitability [7,30]. Vacuoles accumulate high concentrations of Ca^2+^ and sodium (Na^+^), with Cl^−^ acting as a major counteranion. Chloride also plays a prominent role in signal perception and transduction, since a variety of signals (light, pressure, elicitors) cause membrane depolarization by stimulating anion efflux (see Section 3.7).

At the Cl^−^ concentration range usually found in plants of around 50–150 mM of tissue water [5], within the range defined as beneficial (Figure 1C), it represents the dominant inorganic anion in the vacuole, with leaf contents that can be similar to those of the macronutrient K^+^ [2], determining central functions in cell osmoregulatory and turgor-driven processes. It is generally assumed that Cl^−^ serves a non-specific osmotic function and that other anions can provide osmolarity into the plant vacuoles or balance positive charges. However, evidences have recently been provided indicating that Cl^−^ is a quantitatively and qualitatively preferred osmoticum in plants, and cannot be sufficiently replaced by other anionic macronutrients [2,4]. Given that Cl^−^ is not assimilated throughout anabolic metabolism, its accumulation efficiency into shoot tissues of tobacco plants is four times higher than the accumulation of NO_3_^−^, and three times higher than the sum of sulphate (SO_4_^2^^−^) + phosphate (PO_4_^3^^−^) anions, determining more negative osmotic potential and higher turgor [2].

Cells regulate water movement and water-holding capacity by controlling the movement and accumulation of ions. This is constrained by both osmotic and charge balance, and is driven by ion and voltage gradients and by active ion transport. The osmolarity of biomolecules sequestered inside a cell creates a pressure that can easily reach an atmosphere or more in animal cells [31]. Given the absence of cell walls, animal cells must equilibrate internal and external osmolarity. Since the net charge of biomolecules is negative, the osmotic equilibrium is maintained through the reduction of the intracellular Cl^−^ concentration. This is achieved by the driving force of the plasma membrane (PM) electric potential (*E*_m_), negative inside, generated by the Na^+^/K^+^-ATP_ase_ [31]. Therefore, the internal Cl^−^ concentration is of crucial importance to regulate cell osmolarity in animal cells. Computational modeling indicates that in the absence of Cl^−^ conductances, the transmembrane (TM) movements of cations are always osmotically balanced. When a Cl^−^ conductance is present, the only way to keep the Cl^−^ equilibrium potential in accordance with the changed *E*_m_ is to adjust cell volume. Thus, while cations are primarily responsible for the *E*_m_, the Cl^−^ conductance determines the extent of water movement and cell volume changes [32]. The participation of Cl^−^ in controlling osmoregulation and water balance is also relevant in plant cells. Unlike animal cells, plant cells do not live submerged in saline plasma that maintains homogeneous concentrations of Cl^−^ and Na^+^. Extracellular levels of these ions in plant tissues depend on their concentrations in the rhizosphere, so instead of a Na^+^/K^+^-ATPase, plant cells possess H^+^-ATPases in the PM. In addition, the control of water transport is also different in plant cells because of the external cell walls and turgor. To generate turgor pressure, plant cells have a higher intracellular than extracellular osmolarity. The large hypertonic vacuole is the plant cell organ that regulates osmolarity, water accumulation, and cell turgor. Therefore, contrary to animal cells, cytoplasmic Cl^−^ exclusion in plant cells occurs mainly by vacuolar compartmentalization. This may be the reason why Cl^−^ specifically stimulates the vacuolar proton-pumping V-type ATP-ase [33], whereas the PM H^+^-ATP_ase_ is stimulated by monovalent cations, particularly K^+^ [12]. Therefore, Cl^−^ fluxes through the PM and tonoplast is also an essential regulator of intracellular osmotic potential, turgor, and cell volume regulation in plant cells [34]. Not only because of its role in salt partitioning during turgor normalization, but also because Cl^−^ efflux through anion channels depolarizes the PM [35,36,37]. Given that the equilibrium potential for Cl^−^ ions is normally positive (cytosolic concentration exceeding extracellular concentration), activation of Cl^−^ channels results in strong efflux of Cl^−^ from plant cells, leading to PM depolarization. This depolarization activates, in turn, outward rectifying K^+^ channels, which determines a positive feedback for a massive discharge of salts and water. Indeed, anion fluxes have been described as the "pacemaker" of plant cell turgor control [38]. Consistent with the role of Cl^−^ in the regulation of water transport and cell volume in plants, Cl^−^ fluxes are specifically required for the proper functioning of specialized motor cells like the pulvini of leguminous plants during seismonastic and nystinastic leaf movements [39,40,41], and guard cells during stomatal opening and closure [42,43]. Osmotically-driven water fluxes are also essential for endogenous plant organ movement or circumnutation [44]. While transmembrane ionic fluxes necessarily involve electroneutral transport with K^+^, the activation of PM anion channels is often the triggering element that connects environmental cues with the response of the corresponding motor cell, as observed in the stomatal closure or the circadian-regulated leaf opening [45,46].

**Figure 3:**
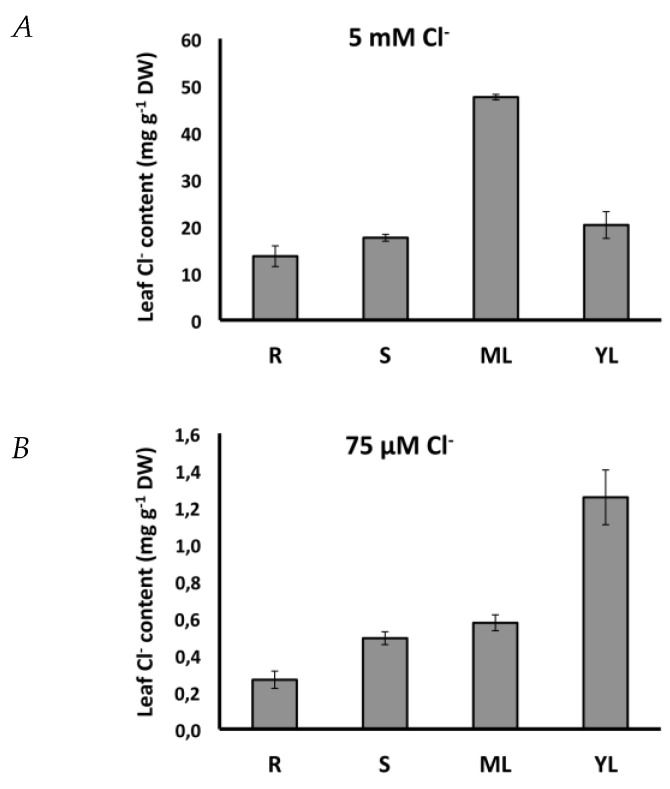
Preferential accumulation of Cl^−^ in growing tissues. When available at macronutrient levels (**A**), Cl^−^ is distributed throughout the plant, reaching its maximum concentration in adult leaves, where it is stored in their large vacuoles. When present at lower concentrations, sufficient to meet micronutrient requirements but insufficient as a macronutrient (**B**), tobacco plants prioritize preferential Cl^−^ accumulation in actively growing young leaves, indicating a biological role in plant cell growth. Colmenero-Flores et al., unpublished data.

As a result, Cl^−^ is more efficient in providing cell osmolarity, water accumulation, and turgor, the driving force of plant cell elongation. Then, is Cl^−^ specifically well suited to stimulate cel elongation? When available at macronutrient levels, Cl^−^ is distributed throughout the plant, reaching its maximum concentration in adult leaves, where it is stored in the large vacuoles (Figure 3A). However, when present at lower concentrations, sufficient to meet micronutrient requirements but insufficient as a macronutrient, tobacco plants prioritize preferential Cl^−^ accumulation in actively growing young leaves (Figure 3B), indicating a biological role in plant cell growth. Micronutrient nutrition is important to ensure adequate cell division rates [47] but, at higher concentrations, Cl^−^ is specifically required to stimulate leaf cell elongation in tobacco plants [2]. Chloride-dependent stimulation of plant cell growth has been demonstrated in other organs and cell types like: Epidermal cells from elongating internodes of *Pisum sativum* [48]; cells of elongating coleoptiles of grass seedlings [49,50,51]; the elongating stigma of grasses at the onset of flower anthesis [52]; and during pollen tube elongation [34,53,54,55]. For some of these processes, its substitution by NO_3_^−^ or other inorganic anions avoids or reduces the stimulation of cell elongation [2,51], revealing Cl^−^ specificity. In fact, strong stimulation of leaf expansion and plant growth resulting from NO_3_^−^ application (e.g., from 5 to 10 mM NO_3_^−^) is a consequence of an increased rate of cell division, but not of cell elongation, which specifically requires Cl^−^ [2]. Interestingly, the stimulation of leaf cell size in tobacco plants occurred with Cl^−^ treatments as low as 150 μM Cl^−^ [2]. This treatment hardly increased leaf Cl^−^ concentration by 4 mM, suggesting that besides the osmotic effect, a specific signaling role of Cl^−^ on the stimulation of leaf cell growth [2]. Most of the processes where Cl^−^ stimulates cell elongation or the functioning of motor cells have been proved responsive to auxin [51], which in turn stimulates cell Cl^−^ uptake [40,48,49] as a prerequisite for cell elongation. The mechanisms that regulate the interactions between plant development, cell cycle, phytohormone activity, and chloride homeostasis in plants are unknown.

Besides the physiological evidence, genetic approaches have revealed the relevance of Cl^−^ homeostasis in cell elongation (see Section 4.3). Thus, disruption of *DTX33* and *DTX35* genes, encoding vacuolar channels involved in Cl^−^ compartmentalization during cell expansion, resulted in shorter root hairs and defective pollen-tube growth. In addition, lines of different plant species mutated in the gene encoding cation-chloride cotransporter (CCC) proteins, involved in the regulation of Cl^−^ homeostasis in animal and plant cells, display severe alterations in the elongation of different cell types such as the elongating region of the inflorescence stem of *A. thaliana* [56], and the elongating root cells of rice [57].

### 3.2. Cell Water Balance and Tissue Hydration

The stability of water molecule interaction is atypically high in the solvation shell of halogen anions [58,59], making Cl^−^ an osmolyte with uncommon physical properties, very suitable to enhance the retention of water. Chloride is also the anion that generates the lowest density solution of any of the biologically relevant anions considered by Boyd and Gradmann [60]. Taken together, this and the previously described properties of Cl^−^, promoting higher osmolarity, larger leaf cells with higher ability for ion compartmentalization, and higher turgor, it is expected that macronutrient Cl^−^ nutrition increases the water storage capacity of plant cells. In agreement with this proposition, Cl^−^ accumulation to macronutrient levels specifically increases water content, relative water content, and succulence in the leaf [2], confirming the traditional role attributed to Cl^−^ in favoring the hydration of plant tissues [5,6].

### 3.3. Whole-Plant Water Relations, Photosynthesis, and Water-Use Efficiency

**Figure 4:**
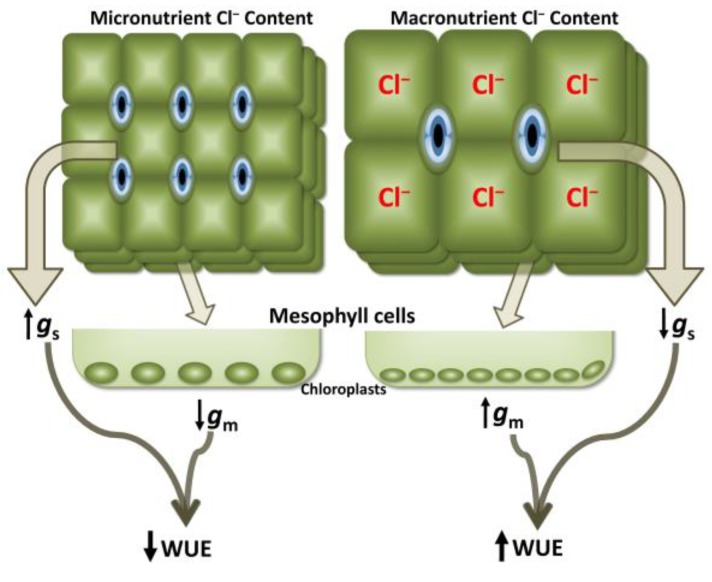
Chloride (Cl^−^) nutrition at macronutrient levels significantly increases the size of leaf cells, resulting in a reduction in stomatal density and, therefore, conductance (g_s_). At the same time, Cl^−^ improves mesophyll diffusion conductance to CO_2_ (*g*_m_), due, at least in part, to increased surface area of chloroplasts exposed to the intercellular airspace. The higher mesophyll diffusion conductance compensates for the reduction in stomatal conductance, resulting in overall higher WUE [23]. Upward arrows indicate higher values, and downwards indicate lower values. Figure obtained from Maron, 2019 [61].

A recently reported and unexpected effect of Cl^−^ nutrition on the physiology of tobacco plants is the reduction of leaf transpiration as a consequence of a lower stomatal conductance (*g*_s_; [2]). This effect was not a consequence of a lower stomatal opening, but resulted from the reduction of the stomatal density associated to the higher enlargement of leaf cells in Cl^−^-treated plants [23]. Therefore, Cl^−^ simultaneously stimulates growth and reduces water consumption, which results in a clear improvement of water-use efficiency (WUE; [2,23]). Interestingly, the reason why a lower *g*_s_ does not result in lower photosynthetic capacity (as expected for C_3_ plants) is because Cl^−^ specifically increases the mesophyll diffusion conductance to CO_2_ (*g*_m_; [23]). This phenomenon is associated, at least in part, with a higher surface area of chloroplasts exposed to the intercellular airspace of mesophyll cells, pointing to a role of Cl^−^ nutrition on chloroplast performance (see Section 3.6). The higher *g*_m_ compensates for the reduction in *g*_s_, resulting in overall higher WUE (Figure 4). Increasing crop yields, while also improving WUE, has become a major focus of plant research. The beneficial effect of macronutrient Cl^−^ levels in maintaining high photosynthesis rates while improving WUE is particularly challenging in C_3_ plants, in which water loss through transpiration is inherent to the process of fixing atmospheric CO_2_.

Improvement of WUE as a result of water balance regulation by Cl^−^ nutrition, at both the cellular and whole-plant levels, probably responds to adaptive mechanisms that increase the ability of plants to withstand water deficit, a hypothesis that deserves further investigation.

Correlations between root hydraulics (water flow) and K^+^ [62] or NO_3_^−^ [63,64] ions homeostasis have been established. Also, a positive correlation between water use and Cl^−^ transport has been reported when comparing citrus genotypes with contrasting capacity of Cl^−^ inclusion [65,66]. However, the specific effect of Cl^−^ on root hydraulic conductivity, through regulation of aquaporin-mediated water transport across the PM of root cells, is an issue still unknown. According to its relevance in the regulation of plant osmolarity and water flux, a role in maintaining xylem volume flow and root pressure has been reported for both K^+^ and Cl^−^ ions [12]. Additionally, a role of Cl^−^ in facilitating loading and unloading of sugars in the phloem sap has been also proposed [67,68].

### 3.4. Energy Efficiency and Increase of Dry Biomass

The increase of biomass production induced by macronutrient Cl^−^ nutrition is associated with the stimulation of higher turgor, cell size, and shoot expansion (see Section 3.1). However Cl^−^ not only increases fresh weight as a consequence of greater water accumulation, but it also promotes higher dry biomass [2]. This phenomenon requires an effective increase in the efficient assimilation of both carbon and N. The most abundant anionic species used as osmoregulatory molecules in plants are Cl^−^, NO_3_^−^, and malate. Considering the energy cost, Cl^−^ accumulation is particularly efficient. On the one hand, the energy cost of generating osmolarity by Cl^−^ influx is invariably less than that of synthesizing organic acids [3]. On the other hand, the cost of vacuolar compartmentalization of Cl^−^ might require less energy in comparison to NO_3_^−^ sequestration [4]. The proposed reason is that the prevalent transport mechanism for Cl^−^ compartmentalization is based on the activity of anion channels [69,70], whereas NO_3_^−^ depends to a higher extent on secondary active transport mechanisms [71]. In addition, compartmentalization of Cl^−^, a highly available and "cheap" osmolyte, reduces the vacuolar accumulation of malate and NO_3_^−^, making these important sources of carbon and N more available for plant metabolism and growth [2,6]. The calculated contribution of organic anions to balance the positive charge of inorganic cations in plants treated with 10 mM NO_3_^−^ was reduced 2.6 times in plants treated with 5 mM NO_3_^−^ + 5 mM Cl^−^ [2]. Supporting the role of Cl^−^ in preventing the diversion of important sources of N and carbon, halophyte plants growing under Cl^−^-deficient conditions accumulate higher concentrations of NO_3_^−^ [72,73] and malate [74] than those plants growing under optimal sodium chloride (NaCl) concentrations. Apart from these considerations, it is expected that all the benefits associated with macronutrient Cl^−^ nutrition, such as better water status, greater mesophyll diffusion to CO_2_, and possibly other factors yet to be described, improve plant development, metabolism, and growth.

### 3.5. Cl^−^/NO_3_^−^ Interaction and Nitrogen-Use Efficiency

Nitrogen is the most limiting nutrient for the growth of land plants, essential for protein, nucleic acid, and secondary metabolism. Nitrate is one of the major N sources, as well as a signal molecule involved in the control of many physiological processes, plant growth, and crop yield [75,76,77]. Both NO_3_^−^ and Cl^−^ are the most abundant inorganic anions in plants and share similar physical properties and TM transport mechanisms, which is in the origin of the strong dynamic interactions between the two monovalent anions [4]. Root Cl^−^ uptake is apparently inhibited in the presence of external NO_3_^−^ [78,79]. Similarly, a higher accumulation of Cl^−^ leads to lower NO_3_^−^ content in plants, suggesting that influx of the two anions could be facilitated by the same transport mechanisms [5,80]. This antagonistic interaction between Cl^−^ and NO_3_^−^ is one of the reasons why Cl^−^ is considered harmful to agriculture, with many publications reporting a detrimental competition between Cl^−^ and NO_3_^−^ in many crops (reviewed in [5]). The terms by which this competition is generally explained point to a strong preference for NO_3_^−^ over Cl^−^ in plants. It is a reasonable strategy, given the nutritional importance of NO_3_^−^, a molecule that after root uptake is preferentially used for anabolic assimilation, whereas Cl^−^ accumulates in plant tissues because it is not metabolized. In addition, if the net Cl^−^ uptake rate is substantially higher than the dilution rate determined by the plant growth, progressive increase of Cl^−^ concentration may eventually damage sensitive plant organs. The different types of selectivities explaining how these two anions are discriminated by proteins have been recently described in detail [4], and more recent findings will be addressed in Section 4.1.

Nitrogen use efficiency (NUE), which defines the plant yield per unit of applied N, is an important agricultural trait to reduce excessive use of chemical fertilizers, with substantial benefits to farmers and the environment [81,82]. Nitrate uptake and allocation are key factors regulating NUE [76]. Given the close interaction between Cl^−^ and NO_3_^−^, it is expected that Cl^−^ can significantly influence NUE. The key question is whether Cl^−^ improves NUE or makes it worse. The common belief is that Cl^−^ reduces NUE by constraining not only root NO_3_^−^ uptake, but also root-to-shoot translocation or vacuolar compartmentalization. Different studies have reported a negative effect of Cl^−^ on root NO_3_^−^ uptake [83,84], which is expected to impair NUE. Nevertheless, net NO_3_^−^ uptake results from the difference between NO_3_^−^ influx mediated by active transport and its passive efflux through anion channels. Root anion efflux to the rhizosphere might be important to regulate H^+^-ATPase activity, maintaining the H^+^ charge balance [85], or to regulate plant cell growth [86]. The release of Cl^−^ from root cells through anion channels, replacing NO_3_^−^ efflux, could be an important mechanism to prevent the loss of such an important source of N [4], which is expected to improve NUE. Vacuolar compartmentalization of NO_3_^−^ determines the extent to which this nutrient can be accumulated in plant tissues, and different studies have revealed a close relationship between NO_3_^−^ content and NUE. Thus, the lower compartmentalization capacity of the *A. thaliana* knockout line *aha2/aha3*, with lack of vacuolar ATPase function, deals to 80% reduction in NO_3_^−^ storage capacity and severe growth reduction [87]. In a *Brassica napus* variety, increased NUE correlates with higher shoot and lower root NO_3_^−^ accumulation [81]. According to the nutrients diversion hypothesis described in Section 3.3, we could expect a different scenario. Thus, the idea underlying the preferential compartmentalization of Cl^−^ points to plants using Cl^−^ as a preferred osmoregulatory molecule, while NO_3_^−^, an essential nitrogen source for land plants, should be preferentially assimilated and used as an osmolyte when Cl^−^ is not sufficiently available in the soil [2], or as a result of high NO_3_^−^ content in the external medium [88,89]. The addition of to a basal nutrient solution of a salt supplement containing 5 mM Cl^−^ reduced leaf NO_3_^−^ content of tobacco plants by 6.5 times, whereas the supplementation with salts containing equivalent amounts of phosphate + sulphate salts reduced leaf NO_3_^−^ content by 1.7 times [2]. Interestingly, although Cl^−^-treated plants contained 3.6 times less NO_3_^−^ concentration than phosphate + sulphate plants, they had higher biomass. This strongly suggests that Cl^−^ improves NUE, despite significantly reducing foliar NO_3_^−^ storage. This would represent a radical change in the perception of Cl^−^ from a NO_3_^−^ antagonist to a nutrient that promotes a more efficient use of N. To summarize, the most likely scenario proposed is the following. When NO_3_^−^ is available, active transport mechanisms (frequently more selective for NO_3_^−^ than for Cl^−^; see Section 4.1) prioritize NO_3_^−^ influx by inhibiting Cl^−^ uptake [78,90]. When little NO_3_^−^ is available, Cl^−^ influx is less inhibited, increasing root uptake and intracellular Cl^−^ concentration, which is expected to replace NO_3_^−^ in serving an osmotic function, allowing a more efficient use of the available N. Species or varieties with higher Cl^−^ inclusion capacity (e.g., tobacco) may have anion influx transporters with higher Cl^−^ selectivity (see Section 4.1). Nevertheless, direct evidence is still required to clarify whether NUE is favored by either efficient NO_3_^−^ or Cl^−^ compartmentalization in shoot tissues.

Another aspect that may suggest an interaction between Cl^−^ and NUE is the stimulatory effect of Cl^−^ on the asparagine synthetase activity. Chloride increases the affinity of asparagine synthetase for glutamate, its substrate [14]. Asparagine is a major compound in the long-distance transport of soluble N in many plant species, suggesting a role of Cl^−^ nutrition in N metabolism and transport [12].

### 3.6. Chloroplast and Organellar Performance

Chloride is the most abundant anion in the chloroplast stroma (50–90 mM Cl^−^) [91]. After onset of illumination, Cl^−^ influx from the stroma to the lumen is essential for thylakoid swelling. Conversely, Cl^−^ re-export to the stroma would cause the thylakoid to shrink during transition to darkness [92,93]. Similar to the role played by Cl^−^ in promoting cell turgor and elongation [2,4], regulation of thylakoid swelling suggests a possible role of Cl^−^ in promoting chloroplast osmoregulation and growth, which could be a prerequisite for its subsequent division [94]. Thus, increased mesophyll diffusion to CO_2_ in plants treated with Cl^−^ at macronutrient levels is apparently related to a greater biogenesis of chloroplasts [23]. Chloride fluxes also play important roles in the regulation of photosynthetic electron transport and photoprotective mechanisms in chloroplasts (see Section 4.3). The accumulation of protons in the thylakoid lumen is electrically counterbalanced by Cl^−^ influx [92,93,95], suggesting that Cl^−^ regulates the generation of the pH gradient between the lumen and the stroma [11]. Adequate Cl^−^ homeostasis, regulated by thylakoid Cl^−^ channels (see Section 4.3), is required to adjust photosynthesis to fluctuating light and environmental conditions [96,97,98,99,100]. Therefore, Cl^−^ is important for the proper functioning of the chloroplast, introducing an element of specificity (e.g., it requires Cl^−^-specific channels) that, at least in part, may explain the requirement of Cl^−^ over similar molecules such as NO_3_^−^ [23]. Recent data in animal cells indicate a crucial role of Cl^−^ transport in organellar physiology that goes beyond the electrical shunt required for acidification of cellular organelles [101]. Other relevant functions in animal cells point to the regulation of adequate endocytosis and lysosomal storage, as well as membrane traffic by regulating organellar ion homeostasis and osmolarity [101,102]. In contrast, the role of Cl^−^ in vesicular trafficking and function remains unexplored in plant cells.

### 3.7. Other Functions: Electrical Signals, Circulating Ion Currents, and Plant Immunity

Specific roles of Cl^−^ in propagating electrical signals, and in circulating ion currents have also been described, although these functions do not apparently require macronutrient Cl^−^ levels. Electrical signals, including action potentials, variation potentials, slow wave potentials, and system potentials, are rapidly propagated in response to both biotic and abiotic stimuli, and are defined as an ion imbalance across the PM, leading to a voltage transient [103,104,105]. Ion fluxes circulating through pollen tubes, involving both Cl^−^ uptake in mature zones and Cl^−^ release at the growing apex, are important for pollen germination and pollen tube growth [53,106].

Activation of R-type anion channels has been proposed as an essential step for triggering the ROS-dependent plant innate immune response [107]. Besides its function in chloroplast Cl^−^ homeostasis, the *A. thaliana* thylakoid Cl^−^ channel AtCLCd negatively regulates plant immunity, whereas elicitors regulate in turn the *AtCLCd* gene expression [108]. Interestingly, diuretic compounds, which are specific inhibitors of animal and plant cation/Cl^−^ cotransporters, prime plant immunity in *A. thaliana* [109]. Further research is required to better understand the interaction between Cl^−^ homeostasis and the plant immunity response. 

### 3.8. Relevance of Cl^−^ for Crop Yield

The idea that only small amounts of Cl^−^ are required for optimal plant growth and that naturally occurring Cl^−^ levels amply meet crops requirements still underlies the agronomic—and even the scientific—fields [10]. However, according to the findings described above, crops could benefit from Cl^−^ fertilization more broadly than is generally believed. The amount of Cl^−^ fertilization required to ensure beneficial macronutrient requirements would depend on the levels naturally present in the soil and on the specific necessity of the cultivated crop. In inland regions, far from the ocean, the low deposition of Cl^−^, a highly mobile molecule subject to leaching in the soil, can limit the yield of crops [110]. Substantial responses to Cl^−^-containing fertilizers have been reported for different crops in many parts of the world [5,111]. However, most of these studies did not clarify to what extent plant yield enhancement was due to the accompanying cations, or whether other anions could replace Cl^−^ in such a growth-promoting effect. It has been recently proven that a number of physiological disorders impairing the growth and yield of durum wheat under field conditions are specifically due to soil Cl^−^ deficiency [112]. 

For reasons still unknown, some plant species such as kiwi fruit [113] and palm trees [114] have higher Cl^−^ requirements, which cannot be alleviated through NO_3_^−^ addition. These plants can be valuable models for better understanding of the regulation of Cl^−^ homeostasis in higher plants [4]. For example, coconut plants appear to have greater dependence on Cl^−^ for proper regulation of stomatal function, since stomatal opening is delayed by about 3 h in Cl^−^-deficient plants [5]. Interestingly, guard cells of another palm tree, *Phoenix dactilifera*, release Cl^−^ rather than NO_3_^−^ during stomatal closure, while NO_3_^−^ is required as a signal molecule to trigger the abscisic acid (ABA)-dependent response [115]. This clearly demonstrates that full understanding of Cl^−^ homeostasis in higher plants requires going beyond of model plant species. Watanabe et al. [116] reported leaf Cl^−^ concentrations for 670 species from 138 families of terrestrial seed plants collected from their natural habitats. The most frequent plant Cl^−^ content reported was around 5 mg g^−1^ DW, which is below the beneficial range of Cl^−^ nutrition (Figure 1C). This suggests that plants might frequently benefit from Cl^−^ fertilization in many environments. In the agronomic context, Cl^−^-deficient soils can be identified in terms of plant growth for important crops like coconut, oil palm, wheat, durum wheat, and maize (reviewed in Xu et al. [5] and Raven [3]). Therefore, these—and most probably other—species are favored by Cl^−^ fertilization, which is expected to improve plant performance and crop yield.

In addition, given the close correlation between Cl^−^ homeostasis and NUE (see Section 3.5) adequate management of optimal NO_3_^−^/Cl^−^ ratios in different agriculture systems could reduce NO_3_^−^ input rates without compromising plant performance [117]. Chloride-dependent reduction of plant NO_3_^−^ accumulation in vegetables could also be used as a strategy to decrease excessive NO_3_^−^ content. Vegetables are classified as NO_3_^−^ accumulators [118] and the NO_3_^−^ metabolic derivatives nitrite and nitrosamines are well-known risk factor for human health [119].

### 3.9. Cl^−^ and Salinity

Salt stress conditions produced by high levels of NaCl in the medium can be a serious problem in plant species with little capacity to exclude Cl^−^ from photosynthetic tissues. Typical examples of Cl^−^-sensitive crops are citrus and grapevine. Many works dealing with this topic have been published in the last 30 years. Recently, different types of anion transporters have been characterized that can be relevant in the ability of plants to exclude Cl^−^. This issue has been addressed in an important number of recent publications and reviews [8,9,11,80,120,121,122], which is the reason why it has not been included here.

As a synopsis of this section, a table summarising major breakthrough regarding Cl^−^ function research in plants is given in Table 2. Many relevant findings have been described during the last ten years.

## 4. Regulation of Cl^−^ Homeostasis

Although the pathways for root Cl^−^ entry and movement within the plant have been biochemically and electrophysiologically characterized [7,125], their molecular determinants are poorly defined. The recently raised interest on Cl^−^ homeostasis has been accompanied by the identification and functional characterization of genes involved in the transport of this nutrient [4,8,11,80,120,125]. The interest has focused more on the study of Cl^−^ exclusion mechanisms, important for salinity resistance [8,80], while transport mechanisms relevant to Cl^−^ nutrition are less understood. Guard cell membrane anion transport systems and their regulatory components have been exhaustively characterized and do not present great relevance in the regulation of Cl^−^ nutrition, so they will not be addressed in this review. We refer the reader to recent reviews that address and update this topic [126,127,128].

### 4.1. Cl^−^ Influx and Net Cl^−^ Uptake in the Root

Under external Cl^−^ concentrations relevant for Cl^−^ nutrition in the micro- and macronutrient ranges (Figure 1), the symplastic pathway dominates root Cl^−^ uptake and transport in plants [18,129,130,131,132,133,134,135,136]. This point has been demonstrated in citrus and grapevine varieties with contrasting capacities to accumulate Cl^−^ [18,136]. Using a Cl^−^ concentration within the beneficial range (4.5 mM Cl^−^), it is shown that both active and poor Cl^−^ includer citrus rootstocks modulated Cl^−^ influx according to the availability of the nutrient, since uptake capacity was induced by Cl^−^ starvation but inhibited after Cl^−^ re-supply. And both active and poor Cl^−^ includer rootstocks take up Cl^−^ to much higher concentrations than those needed to fulfil micronutrient requirements [18]. Therefore, regulation of Cl^−^ transport and accumulation resemble that of a nutrient like K^+^, instead of that of a toxic element like Na^+^. For instance, the efflux to influx ratio of Cl^−^ and K^+^ across the PM of plant cells is rather similar, whereas the ratio for Na^+^ is normally higher [22]. The fact that Cl^−^ applications in the 1–5 mM Cl^−^ range determine increasing values of leaf Cl^−^ accumulation in a linear fashion [2], which in turn determine positive responses in terms of dry biomass (Figure 2), strongly supports that plants regulate the required amount of Cl^−^ within the beneficial macronutrient range. Regulation of Cl^−^ transport is adjusted through changes in the maximum transport capacity and the affinity for Cl^−^ [137,138,139].

In plant cells, net Cl^−^ uptake results from combined activities of influx and efflux transporters [22,50]. The principal driving force for Cl^−^ flux across the PM is its electrochemical gradient. Since Cl^−^ is a negatively-charged molecule, and taking into account its cytosolic concentration around 10–15 mM [80,140,141], Cl^−^ equilibrium potential is much lower than the resting potential of the PM under non-saline conditions. As a consequence, under most circumstances, Cl^−^ influx requires active transport that drives Cl^−^ against the electrochemical gradient through high- and low-affinity Cl^−^/H^+^ symport mechanisms [21,50,133,137,142,143,144]. Cell Cl^−^ release is, however, down the electrochemical gradient and mediated by anion channels [7,22,125]. Passive Cl^−^ uptake through anion channels is only possible as a consequence of a strong and rapid increase in soil salinity, when transient PM depolarization permits *E*_m_ to be less negative than the Cl^−^ equilibrium potential. Under these circumstances pH-independent Cl^−^ and NO_3_^−^ influx activities have been registered [140,141,145]. These currents, mediated by anion channels and activated by PM depolarization, are probably important to maintain electroneutral Na^+^ uptake, thereby preventing excessive PM depolarization [125]. However, according to Bazihizina et al. [146], this scenario is unlikely for the majority of Cl^−^ concentrations in field conditions, and active Cl^−^/H^+^ symport mechanisms should dominate root Cl^−^ uptake even in halophyte species up to 600 mM external NaCl.

#### 4.1.1. Cl^−^ Influx

The molecular identity of PM transporters likely to catalyze Cl^−^ influx has been recently revealed [147]. Members of the plant Nitrate transporter 1/Peptide transporter Family (NPF; [148]) have been primarily described as low-affinity active NO_3_^−^ transporters with a H^+^ coupling ratio of 2H^+^:NO_3_^−^. However, many NPF family members also transport several other substrates such as nitrite, glucosinolates, phytohormones, and Cl^−^ [149]. Although a substrate is known for many of the Arabidopsis NPF members, the detailed transport mechanisms and selectivity are not adequately described for most of them [150]. The H^+^-coupled active transport ability requires the ExxER/K motif containing three chargeable residues that can bind protons in the first TM domain [151]. In the model plant *A. thaliana*, the identified NPF proteins capable of transporting Cl^−^, AtNPF2.4, and AtNPF2.5 lack the ExxER/K motif and mediate passive Cl^−^ release [152,153]. In the monocot *Zea mays*, two members of the NPF6 subfamily have been characterized as active transporters involved in H^+^-coupled Cl^−^ influx [147]. These PM active Cl^−^ transporters, ZmNPF6.4 and ZmNPF6.6, are the closest maize homologs of the *A. thaliana* NO_3_^−^ transceptor AtNPF6.3, also known as CHL1 or NRT1.1 [154]. AtNPF6.3 has been characterized as a dual affinity transporter that moves NO_3_^−^ in both the high- and the low-affinity ranges [155,156]. Taking advantage of the AtNPF6.3 crystal structure [157,158], a key role for NO_3_^−^ binding has been proposed to the protonable His356 residue. The maize transporter ZmNPF6.6, which contains the equivalent His362 residue, is a high-affinity NO_3_^−^ transporter that can also transport Cl^−^ in the low-affinity range. Both AtNPF6.3 and ZmNPF6.6 transport Cl^−^ when NO_3_^−^ is absent, and this transport capacity is strongly inhibited by NO_3_^−^, possibly as a consequence of NO_3_^−^ occupation of the substrate-binding pocket [90]. However, the maize transporter ZmNPF6.4, which contains a Tyr370 residue (not protonable at physiological pH) in the equivalent position of the putative substrate-binding pocket, is a high-affinity Cl^−^ selective transporter (not inhibited by increasing external NO_3_^−^ concentrations up to 1 mM) [147]. Interestingly, mutation of the NPF6.4 Tyr-370 to His (Y370H) resulted in change from Cl^−^ selective to high-affinity NO_3_^−^ selective transport activity. Furthermore, the reciprocal mutation in NPF6.6, H362 to Tyr (H362Y) eliminated both NO_3_^−^ and Cl^−^ transport activities. Wen et al. [147] proposed that the His residue is required for the high affinity NO_3_^−^ activity in ZmNPF6.6, while a yet unknown protonable residue is required for high affinity Cl^−^ transport in ZmNPF6.4.

These results indicate that within the NPF family, active high- and low-affinity Cl^−^ transporters occur in different plant species. In *A. thaliana*, which may have a lower Cl^−^ requirement (Table 1; Section 4.2), the AtNPF6.3 transporter is highly selective for NO_3_^−^. In maize, which could have a higher nutritional requirement for Cl^−^ according to Section 3.6, besides the selective NO_3_^−^ ortholog ZmNPF6.6, an additional protein homolog is present, ZmNPF6.4, which is a high-affinity Cl^−^ selective transporter [147]. It could be interesting to analyze the amino acids present in the substrate-binding pocket of NPF6 homologs in plant species that can be considered Cl^−^ includers, some of them classified as Cl^−^-sensitive plants due to their greater accumulation capacity. Thus, in tobacco (*N. tabacum*), citrus (*C. clementina*), grapevine (*V. vinifera*), and sunflower (*H. annus*) species, the phylogenetic equivalents of AtNPF6.3 contain, in all cases, a tyrosine in their putative substrate-binding pocket. This raises two questions: (i) Whether the absence of histidine and/or the presence of tyrosine in the substrate-binding pocket can serve as a predictor of the Cl^−^ transport ability in proteins present in the AtNPF6.3 phylogenetic branch; and (ii) if this is the case, does it make sense of the absence of an NO_3_^−^-selective NPF6 ortholog in the aforementioned Cl^−^-includer species? While the role of ZmNPF6.4 in active Cl^−^ influx has been clearly established, direct demonstration that it is a component of the plant root Cl^−^ uptake system deserves further investigation.

Cation Cl^−^ Cotransporter (CCC) proteins catalyze the co-ordinated symport of K^+^, Na^+^ and Cl^−^, and are inhibited by the ‘loop’ diuretic bumetanide, a specific inhibitor of vertebrate 2Cl^−^:Na^+^:K^+^ cotransporters [56]. In the halophyte *Suaeda maritima*, 100 μM bumetanide halved root Na^+^ concentrations, suggesting a possible direct or indirect role of CCCs in regulating root Na^+^ and Cl^−^ uptake [159].

#### 4.1.2. Cl^−^ Efflux

A second component regulating net Cl^−^ uptake is its release from root epidermal and cortical cells down the electrochemical gradient through anion channels, or other passive transport mechanisms [7,22,125,160]. There are excellent reviews describing PM anion channels, describing their kinetic properties and their functions in plants [7,125,160,161,162]. Chloride release has been proposed to make a considerable contribution to the resultant net Cl^−^ uptake at *E*_m_ values more negative than −50 mV [50], although anion channels involved in fine-tuning of cytosolic Cl^−^ concentration (e.g., to modulate Cl^−^ nutrition in plants) have not been clearly identified yet. Electrophysiological studies revealed high expression of depolarization-activated anion efflux channels in the PM of epidermal root hair and cortical cells of *A. thaliana* [163,164]. These channels exhibit the following properties: (i) Fast activation/deactivation kinetics; (ii) strong voltage-dependent activation; (iii) high permeability for sulphate, Cl^−^, and NO_3_^−^; and (iv) modulation by extracellular anions. These properties are typical of the rapid (R-type) anion channel conductances [165,166]. The ALuminium-Activated Malate Transporter ALMT family encodes R-type anion channels in plants. The maize PM channel ZmALMT1 (selective to sulphate, Cl^−^, and NO_3_^−^) is predominantly expressed in mature root tissues and has been proposed to regulate mineral nutrition in plants [167]. The other type of depolarization-activated anion channel, also strongly modulated by the external anion activity, is the slowly activating (S-type) channel, which exhibits other kinetic properties: (i) Weakly voltage-dependent; (ii) does not inactivate; and (iii) higher NO_3_^−^ selectivity [125,168,169]. S-type channels belong to the SLow-type Anion Channel Associated/SLAC1 Homologues (SLAC/SLAH) protein family in plants [170,171]. No members of either the ALMT or the SLAC/SLAH families have been clearly involved in the regulation of net Cl^−^ or NO_3_^−^ uptake in plants. However, Planes et al. [86] have reported a role of SLAH3 in ABA-induced Cl^−^ release from root epidermal cells. SLAH3 activation by ABA is part of a growth inhibition mechanism in germinating seeds of *A. thaliana*. SLAH3-dependent Cl^−^ currents were measured with microelectrodes in root epidermal cells, indicating the possible implication of S-type channels in regulating net Cl^−^ uptake in plants. This finding could be related with the previous observation of Dauphin et al. [172], who described large S-type anion currents strictly induced by severe water deficit in the PM of root hairs from *A. thaliana*. These results are consistent with the ABA-dependent activation of SLAH3 in guard cells [162], but not with the inhibition of anion channels involved in the xylem loading of anions (Section 4.2), also dependent on ABA. This points towards a clear role of SLAC/SLAH (and possibly ALMT) anion channels in the regulation of Cl^−^ nutrition in higher plants. However, identification of the specific transporters involved in this process and how they are regulated by developmental or environmental cues deserve more investigation.

Anion efflux activity can also be regulated by proteins of the NPF family (Section 4.1). Within the NO_3_^−^ excretion transporter subfamily NPF2, the transporter NPF2.5 is involved in excretion of Cl^−^ out of root cells under conditions of salt stress [153], which is expected to enhance Cl^−^ exclusion and salinity resistance. The efflux of anions is also important to regulate root cell pH by electrically counterbalancing the efflux of protons from the cytosol, as demonstrated for the Nitrate EXcretion Transporter1 NAXT1/NPF2.7 protein [173]. Enhanced Cl^−^ efflux during acidosis is thought to also play a role in cytosolic pH homeostasis in plants [174].

### 4.2. Root-to-Shoot Cl^−^ Translocation

Once transported into the root symplast, Cl^−^ follows its chemical gradient from cell-to-cell through plasmodesmata towards the PM of the xylem-pole pericycle, from where Cl^−^ is loaded into the xylem vessels and transported to the shoot by following the transpiration stream [11,175,176]. The loading of Cl^−^ into the root xylem is considered to be electrochemically passive, being thus likely facilitated by PM anion channels or carriers [8,80,125,177]. The root-to-shoot xylem translocation pathway is the key rate-limiting step for shoot Cl^−^ accumulation under both moderate external Cl^−^ concentration (e.g., 5 mM Cl^−^) and salt stress conditions [18,121,136,178]. Several types of anion channel conductances have been identified by patch clamp studies in protoplasts of root xylem parenchyma cells of barley [179] and maize [175]: A Xylem Inward Rectifying Anion Channel conductance (X-IRAC), which is activated by PM hyperpolarization; a Xylem SLowly activating Anion Channel conductance (X-SLAC), with slow activating/deactivating kinetics; and a QUickly activating Anion Channel conductance (X-QUAC), which has quick activating/deactivating kinetics. All of them were shown to be permeable to Cl^−^, being also X-SLAC permeable to malate. These conductances may thus represent the most important gates for anion release into the xylem [125]. Thus, the use of the anion channel inhibitor 4,4′-diisothiocyanatostilbene-2,2′-disulfonic acid (DIDS) significantly reduced xylem anion loading in barley seedlings [180,181]. A hallmark of ion channels involved in Cl^−^ and K^+^ xylem loading is a negative regulation by water deficit. Both X-IRAC and X-QUAC conductances are inhibited by Ca^+^ and ABA, which reduce passive NO_3_^−^ and Cl^−^ release from root stelar protoplasts under water deficit conditions [175,179].

**Figure 5:**
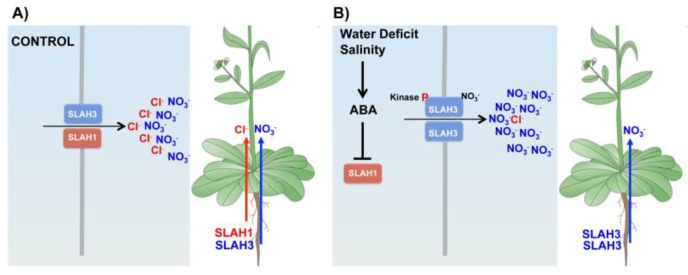
Regulation of root xylem chloride (Cl^−^) translocation by the anion channels SLAH1 and SLAH3 according to environmental cues. Under favorable growing conditions (**A**), high transcriptional activity of both SLAH1 and SLAH3 genes determines the formation of heteromeric SLAH1/SLAH3 complexes in the xylem-pole pericycle. The SLAH1 channel does not transport anions itself, but modifies the kinetic properties of SLAH3, which increases its Cl^−^ conductance by 7 times and mediates xylem translocation of both Cl^−^ and nitrate (NO_3_^−^) anions. However, under abiotic stress conditions like water deficit or salinity (**B**), gene expression of AtSLAH1 is strongly inhibited by an abscisic acid (ABA)-dependent regulatory pathway. This favors the formation of SLAH3/SLAH3 homomers, which significantly reduces the Cl^−^ conductance of SLAH3, decreasing xylem Cl^−^ translocation but maintaining xylem NO_3_^−^ translocation. The schematic representation describes the regulatory mechanism described by Cubero-Font et al. [176].

Recently, Cubero-Font et al. [176] demonstrated the role of two S-type channels, AtSLAH1 and AtSLAH3, in the regulation of xylem loading of Cl^−^ in *A. thaliana*. Gene expression activity of both *AtSLAH1* and *AtSLAH3* genes was found to co-localize in the root xylem-pole pericycle. AtSLAH3 is not active per se, rather it requires extracellular NO_3_^−^ and phosphorylation by Ca^2+^-dependent kinases CPKs [45,182,183], exhibiting high selectivity for NO_3_^−^ over Cl^−^ [45]. Interestingly, AtSLAH1 is unable to conduct anions when expressed in *Xenopus* oocytes. However, when coexpressed with AtSLAH3, the resulting SLAH1/SLAH3 heteromer elicits macroscopic Cl^−^ currents that override the kinase and NO_3_^−^-dependent activation of AtSLAH3. The interaction with AtSLAH1 alters the electrical properties of SLAH3, enhancing the Cl^−^ translocation from pericycle cells into the root xylem vessels. Thus, plants lacking functional copies of any of the two S-type channel genes *AtSLAH1* or *AtSLAH3* genes reduced up to 50% the Cl^−^ content in the xylem sap secretion [176]. The AtSLAH1 membrane protein functions like a molecular switch that regulates the degree of NO_3_^−^ versus Cl^−^ conductance according to environmental cues [176]. Under optimal growing conditions, the *AtSLAH1* gene is highly expressed, favoring the presence of the SLAH1/SLAH3 heteromer, which determines a significant increase of the SLAH3 Cl^−^ conductance. This ensures the simultaneous translocation of both NO_3_^−^ and Cl^−^ nutrients in actively growing plants. Under abiotic stress conditions, such as water deficit or salinity, the expression of the *AtSLAH1* gene is strongly repressed in an ABA-dependent manner [176,184], significantly reducing the Cl^−^ conductance of SLAH3, therefore diminishing xylem Cl^−^ translocation (Figure 5; [176]). Candidate gene(s) encoding the X-QUAC type channel, which is supposed to be the dominant conductance for Cl^−^ xylem loading [175,179], is still missing. The PM AtALMT12 channel is a good candidate because of its permeability to Cl^−^ [8,185] and its presence in root stelar cells [186].

The stelar-localized AtNPF2.4 PM protein has also been identified as a transporter that facilitates the root-to-shoot transfer of Cl^−^, regulating its accumulation in the shoot in response to salt stress by the rapid down-regulation of the *AtNPF2.4* gene [152]. In addition, according to their electrophysiological features, the NO_3_^−^ transporters AtNPF7.2 and AtNPF7.3 could also regulate xylem Cl^−^ retrieval and loading, respectively [8]. It should be noted that inward currents elicited by SLAH1/SLAH3, around −10,000 nA [20,176], are significantly higher than those elicited by NPF transporters. When the *Xenopus* oocyte PM was clamped at −100 mV, currents elicited by SLAH1/SLAH3 were 20 times greater than those elicited by *AtNPF2.4* (−500 nA; [152]) and 100 times greater than those elicited by the close homolog *AtNPF2.5* (−100 nA; Section 4.1; [153]). Taking into account that the resting membrane potential of plant cells usually have values between −80 and −200 mV [187], S-type channels have a prominent role in mobilizing large anion currents from plant cells in comparison with NPF transporters. The function of NPF transporters in Cl^−^ excretion from plant cells could be relevant under specific physiological conditions, possibly complementing those of S^-^ and R-type channels. However, this hypothesis requires further testing.

It is worth mentioning the variability of the NO_3_^−^/Cl^−^ selectivity in the root-to-shoot Cl^−^ translocation process when different plant species or varieties are compared [20]. Based on the concentration of anions in the xylem sap, the NO_3_^−^ translocation efficiency is similar in two ecotypes of *A. thaliana* (WS and Col-0) and in tobacco (*Nicotiana tabacum cv* Petit Havana) plants. However, the Cl− translocation efficiency is 5−7 times higher and the NO_3_^−^/Cl^−^ selectivity 5−8 times lower in tobacco plants compared to the *Arabidopsis* ecotypes WS and Col-0, respectively, confirming the variability of the Cl− inclusion/exclusion trait when different glycophyte species are compared [20].

Given the strong expression of *AtCCC* in the root vasculature, the cation Cl^−^ cotransporter protein has been proposed to mediate the electroneutral co-transport of Na+:K+:2Cl^−^ between xylem-associated cells and xylem vessels, possibly promoting Cl^−^ retrieval from the xylem under salt stress conditions [56]. However, the AtCCC localization at the Golgi/*trans*-Golgi network [188], the possible difficulty of removing Cl^−^ from the xylem through the cation-coupled mechanism imposed by stoichiometric restrictions [80], and its strong implication on plant developmental processes have questioned its direct involvement in xylem Cl^−^ retrieval. Despite these uncertainties, other evidences still support a role of CCC in regulating long-distance ion transport: (i) The rice OsCCC1 was mainly localized to the plasma membrane [57]; (ii) complementation of the *Arabidopsis ccc* mutant with the grapevine *VviCCC* gene reduced shoot Cl^−^ and Na^+^ content to wild-type levels after growing plants in 50 mM NaCl [188]; and (iii) pharmacological studies with bumetanide, the diuretic inhibitor of mammalian and plant CCCs, showed a reduction in the Na^+^ and K^+^ efflux from the root stelar tissue in barley [189]. New experiments are required to allow the reconciliation of CCC membrane localization with protein function in order to more clearly establish the biological role of plant CCCs [122]. Similar to the role attributed to CCC in the Golgi/*trans*-Golgi network [188], other endomembrane transporters have been proposed to somehow regulate long-distance ion transport. The Cl^−^ channels AtALMT9, GmCLC1, and GsCLC2, involved in vacuolar Cl^−^ compartmentalization, and the GmSALT3/CHX1 cation/H^+^ exchanger from the endoplasmic reticulum have been proposed to regulate shoot Cl^−^ accumulation and salinity tolerance (see Section 4.3). In particular, the capacity of vacuolar Cl^−^ loading in vascular cells apparently plays a crucial role in controlling whole-plant ion movement rapidly after the onset of salinity [70].

### 4.3. Cl^−^ Compartmentalization and Subcellular Cl^−^ Transport

#### 4.3.1. Vacuolar Transporters

Since the vacuole is the organelle responsible for generating cell turgor, different transport mechanisms that facilitate intracellular compartmentalization have been located in the tonoplast. The vacuole storage capacity, higher than 40 mM Cl^−^ in glycophyte plants [190], regulates intracellular ion homeostasis and influences whole-plant ion accumulation and distribution [70]. Tonoplast DeToXification efflux carrier (DTX)/Multidrug And Toxic Compound Extrusion (MATE) proteins are widely known transporters of organic compounds. Recently, two MATE transporters, AtDTX33 and AtDTX35, have been shown to elicit large voltage-dependent inward Cl^−^ currents across the tonoplast [191]. These two channels are highly expressed in diverse *Arabidopsis* tissues, including root hairs and guard cells, driving anion influx into the vacuole during cell expansion and pollen tube elongation [191]. The characterization of these channels has enhanced the relevance of Cl^−^ homeostasis in the regulation of osmolarity, turgor, and cell elongation in plants.

Another protein family largely involved in intracellular anion (NO_3_^−^ or Cl^−^) transport is the ChLoride Channel (CLC) family. Different CLC proteins function as anion-selective channels or as anion/H^+^ exchangers. AtCLCa has been characterized as a two-anion/H^+^ exchanger that drives the active uptake of anions into vacuoles of *Arabidopsis* mesophyll and guard cells, with higher selectivity for NO_3_^−^ over Cl^−^ [71]. CLCa plays an important role in the stomatal function, loading anions into the vacuole of guard cells to open stomata in response to light and releasing them during ABA-induced stomatal closure. Inward or outward direction of anion fluxes is regulated through OST1-dependent phosphorylation of the channel [192]. Similarly to AtCLCa, AtCLCb functions as an NO_3_^−^/H^+^ antiporter in the vacuole, with higher selectivity for NO_3_^−^ over Cl^−^ [193]. However, the functional characterization of *clcb* mutant lines is still required to adequately define the biological function of the CLCb transporter. A soybean homolog of the *Arabidopsis* AtCLCa/AtCLCb transporters, GmCLC1, is a vacuolar pH-dependent Cl^−^ transporter (probably a H^+^/Cl^−^ antiporter) that controls shoot Cl^−^ accumulation and NaCl tolerance [194,195,196]. This is another interesting example where the Cl^−^ versus NO_3_^−^ selectivity of an anion transporter varies among species. AtCLCc also localizes to the tonoplast and is possibly involved in vacuolar Cl^−^ compartmentalization in accordance to its selectivity motif [197]. Overexpression of the *AtCLCc* gene leads to increased Cl^−^ accumulation and higher salt stress tolerance in *A. thaliana* [198]. AtCLCc is mainly expressed in guard cells, pollen grains, and roots, with *clcc* mutants unable to regulate the turgor changes in guard cells [199]. Vacuolar GsCLC-c2 from wild soybean (*Glycine soja*) transports both Cl^−^ and NO_3_^−^ with similar affinities, being the affinity for Cl^−^ independent of pH [200]. Similarly to *GmCLC1*, overexpression of *GsCLC-c2* increases Cl^−^ accumulation in the root, improving Cl^−^ exclusion from the shoot and increasing NaCl resistance [200]. *AtCLCg*, the closest homolog of *AtCLCc*, also encodes a vacuolar transporter that is strongly expressed in mesophyll cells, hydathodes, and phloem within the leaf [201]. As AtCLCc, AtCLCg plays a physiological role in plants during salt stress and both genes act in the same pathway through reciprocal control of their expression [201].

Regarding the ALMT family, AtALMT9 mediates Cl^−^ influx into the vacuole of mesophyll cells, guard cells, and roots [69,202]. Baetz et al. [70] have reported high expression of ALMT9 in both the root and shoot vasculature and a possible role in the regulation of long-distance transport of Cl^−^ and Na^+^.

#### 4.3.2. Golgi Transporters

In the Golgi apparatus, the putative H^+^/Cl^−^ antiporters AtCLCd and AtCLCf are present in the trans- and cis-Golgi, respectively, where they have been suggested to participate in luminal pH regulation [203,204]. As explained in Section 3.7, the role of H^+^/Cl^−^ in endosomal and lysosomal membranes of animal cells goes apparently beyond the regulation of pH by counterbalancing H^+^ charges [101,102], but alternative functions still remain unknown in plant cells. The grapevine and *Arabidopsis* CCC localize to the Golgi and Trans-Golgi Network, but the role played by this transporter in this cell compartment is unknown (See Section 4.2).

#### 4.3.3. Chloroplast Transporters

As an essential micronutrient, Cl^−^ plays a necessary function in photosynthesis, stabilizing the oxygen-evolving complex of photosystem-II (see Section 2). At macronutrient levels, Cl^−^ improves chloroplast performance through different mechanisms: (i) Improving the mesophyll *g*_m_ ([23]; Section 3.6); and (ii) as the major counteranion, regulating photosynthetic electron transport [97,98,99,205,206]. It is therefore not surprising that the chloroplast envelope and the thylakoid membrane exhibit a high permeability for Cl^−^ [93,206]. The *Arabidopsis* AtCLCe channel is targeted to the thylakoid membrane of chloroplasts [204,207]. Although AtCLCe was initially reported to be important in maintaining normal cellular NO_3_^−^ levels [161,193], it has also been characterized as a Cl^−^ channel [97]. Considering the amino acid sequence in the anion selectivity filter, CLCe is expected to function as a channel rather than a Cl^−^/H^+^ transporter [97,208]. After transition from light to dark, AtClCe plays a major role in driving Cl^−^ export into the thylakoid lumen [97]. After transition of dark to light, the Voltage-dependent Cl^−^ anion Channel AtVCCN1 (and probably AtVCCN2) [98] fine-tunes the H^+^-motive force across the thylakoid. AtVCCN channels are important for adjusting electron transport and photosynthesis to fluctuating light and environmental conditions [96,98,99,100]. Localization of AtCLCf in the chloroplast envelope has also been proposed [207]. This finding may be compatible with the previously mentioned Golgi localization [100], since a synapse-like interaction between the chloroplast and Golgi body has been reported [209].

#### 4.3.4. Endoplasmic Reticulum Transporters

The soybean cation/H^+^ exchanger of the Cation/Proton Antiporter 2 (CPA2) family named GmSALT3/CHX1 localizes to the endoplasmic reticulum of root vasculature-associated cells. The characterized members of this family are considered to be K^+^/H^+^ exchangers [8]. While GmSALT3 affects Na^+^ exclusion and salt tolerance in soybean [210], it also promotes Cl^−^ exclusion from the shoot [211,212]. Given that GmSALT3/CHX1 are considered to be K^+^/H^+^ exchangers, the mechanism by which they regulate Cl^−^ (and Na^+^) homeostasis remains unknown.

### 4.4. Cl^−^ Redistribution

During plant growth, Cl^−^ is translocated from the root to the shoot via the xylem and, afterwards, redistributed via the phloem. Long-distance transport in the phloem takes place in the sieve tubes, with the sieve element/companion cell complex as the functional unit in angiosperms [213]. The phloem loading of Cl^−^ mainly occurs in the stem and leaves, being transported along the phloem tubes to the sink through an osmotic potential gradient [12,214]. Within the phloem, Cl^−^ is relatively mobile [215] and its recirculation in plants (defined as the ratio of phloem/xylem nutrient fluxes) is about 20%, with the phloem Cl^−^ concentration positively correlating with the Cl^−^ of the nutrient solution [7]. In the phloem sap, Cl^−^ concentration seems to play a role in phloem loading and unloading of sugars [68,216].

Under salinity, ion movement across cellular membranes is tightly regulated, being the efficiency of Cl^−^ transport out of sensitive tissues an important factor that could contribute to salt tolerance [80]. Shoot Cl^−^ unload through the phloem pathway may constitute a major regulatory mechanism controlling leaf Cl^−^ exclusion. However, the Cl^−^ reallocation from shoot-to-root may have little effect in Cl^−^ exclusion since, in citrus, the rootstocks rather than the grafted variety determines the magnitude of Cl^−^ exclusion [18,217,218]. Furthermore, recirculation of ions to the roots via the phloem does not significantly contribute to the reduction of Na^+^ and Cl^−^ content in leaf tissue [177,219]. Nowadays, although different NO_3_^−^ transporters involved in its redistribution within the plant have been identified in *Arabidopsis* plants, the identification of membrane transport proteins involved in Cl^−^ allocation through the phloem remains unexplored so far.

As a synopsis of this section, a table summarising functional properties of Cl^−^ transport proteins potentially involved in Cl^−^ nutrition and/or long-distance transport, is given in Table 3.

## 5. Hormonal Regulation and Signal Transduction of Cl^−^ Nutrition

Very little is known about hormonal regulation and signal transduction processes that control Cl^−^ nutrition at the whole-plant level, including root Cl^−^ uptake, long-distance transport, relocation, and Cl^−^ transport readjustment to improve plant acclimatization to changing environmental conditions. Signaling processes that regulate Cl^−^ homeostasis have been characterized more extensively in stomatal guard cells and, to a lesser extent, in the growing pollen tube, with excellent reviews covering the transporters involved and how they are regulated [127,128,220]. We therefore present only a brief summary according to the information present in the cited reviews. In guard cells, ABA perception by ABA receptors PYrabactin Resistance/PYrabactin-Like or Regulatory Components of ABA Receptor (PYR/PYL/RCAR) inhibits phosphatases from the PP2C and PP2A family. This, in turn, prevents the deactivation of: (i) Ca^2+^-independent kinases (SnRK2.2, SnRK2.3, and OST1); (ii) the Ca^2+^-dependent kinase CIPK23/CBL1; and (iii) anion channels (SLAC1, SLAH3, and ALMT12). ABA responses are accompanied by a rise in cytosolic Ca^2+^ that activates CIPK23/CBL1 or Ca^2+^-dependent kinases, which in turn results in activation of S-type anion channels SLAH3 and SLAC1. Anions efflux depolarizes the PM, thereby activating voltage-gated outward rectifying K^+^ channels for K^+^ efflux. The loss of osmolytes and water initiates turgor-driven stomatal closure. In parallel, stomatal opening is prevented by the deactivation of inward rectifying K^+^ channels through direct interaction of the K^+^ channel with SLAC1 or SLAH3. Pollen tube growth relies on the activity of an overlapping set of anion channels (SLAH3, ALMT12, ALMT13, and ALMT14) controlled by similar Ca^2+^-dependent kinases. Binding of LURE1 to its receptors (MDIS1/MIK1/MIK2 or PRK6/PRK3) probably changes anion channel activity.

ABA signaling has been also proposed to activate AtSLAH3 in the epidermal cells of *A. thaliana* roots [86]. This mechanism that triggers growth inhibition of germinating seeds under abiotic stress involves the core ABA signaling elements described in guard cells: PYR/PYL/RCAR ABA receptors, ABA-inhibited protein-phosphatases from the PP2C family, and ABA-activated protein kinases (SnRK2.2 and SnRK2.3). The kinases directly inhibit the C-terminal domain of the PM H^+^-ATPase AHA2, although it is not clear whether they interact with AtSLAH3. This mechanism is based on the inhibition of the PM H^+^-ATPase resulting in cytosolic pH acidification, and on the decrease in cytosolic K^+^ and Cl^−^ resulting in loss of turgor [86]. Conversely, in maize coleoptile, auxin stimulates the PM H^+^-ATPase [51,221] resulting in pH alkalinization, and also stimulates K^+^ and Cl^−^ influx, probably through inwardly rectifying K^+^ channels and active H^+^/Cl^−^ symport mechanisms, respectively, allowing cell turgor adjustment and elongation [51]. This mechanism is similar to that proposed for turgor recovery in osmotically-stressed *Arabidopsis* epidermal root cells [37,222]. From this information, we can deduce that adequate Cl^−^ nutrition requires an active PM ATPase that favors Cl^−^ uptake through secondary transport. This process would be stimulated by the phytohormone auxin, and inhibited by ABA.

On the contrary, the activity of ion channels from xylem-pole pericycle cells involved in the translocation of K^+^ and Cl^−^ ions into xylem vessels is negatively regulated by ABA. To improve drought and salt stress acclimatization, ABA increases solute accumulation within the root by significantly inhibiting the release of ions into the xylem, but having little effect on ion influx into the root [223,224,225]. In maize root stele, water deficit and ABA down-regulate the activity of ion channels involved in xylem loading of inorganic ions, such as the Stelar K^+^ Outwardly-Rectifying channel SKOR [226,227] and the xylem-associated X-QUAC involved in xylem loading of NO_3_**^−^** and Cl^−^ [175]. This inhibition is apparently mediated by an ABA-dependent rise in free cytosolic Ca^2+^. In addition, transcription in xylem-pole root pericycle cells of the *Arabidopsis AtSKOR* and *AtSLAH1* genes is strongly down-regulated by water deficit and ABA ([176,228] and Figure 5).

## 6. Concluding Remarks and Future Prospects

The levels at which Cl^−^ accumulates in plants, typical of a macronutrient, and the consequent improvement of plant performance have led to its designation as beneficial macronutrient. Tobacco plants with macronutrient Cl^−^ levels display more efficient use of water, nitrogen, and carbon/energy. Significant WUE improvement results from concurrent stimulation of growth and reduction of water use. Despite the Cl^−^-dependent improvement of water balance and water relations, a specific effect of Cl^−^ on root hydraulic conductivity is an issue yet unresolved. WUE enhancement by macronutrient Cl^−^ nutrition probably increases the ability of plants to withstand water deficit, a hypothesis that must be explored. Although increased dry biomass of Cl^−^-treated plants clearly points to a more efficient use of N, direct evidence is still required to confirm whether Cl^−^ improves NUE. It must be also clarified if NUE is favored by an efficient compartmentalization of NO_3_^−^ or as a result of NO_3_^−^ replacement by Cl^−^ in the vacuole. The positive effect of Cl^−^ on chloroplast performance may be due to various factors like the regulation of thylakoid swelling, improved photosynthetic electron transport, and photoprotective mechanisms. More research is required to clarify these issues, as well as whether Cl^−^ stimulates chloroplast biogenesis. Chloride promotes cell elongation as a consequence of better and "cheaper" osmoregulatory and turgor-generating ability. That is apparently the reason why auxin stimulates the influx of Cl^−^ into plant cells and why ABA may have the opposite effect. It is therefore important to confirm these points and to accurately determine the signaling pathways that regulate these processes, as well as the inhibition by ABA of ion xylem translocation. We expect that many crops could benefit from Cl^−^ fertilization to a higher extent than previously believed, resulting in the improvement of crop performance, stress resilience, and yield. Further research is also required to clarify the role of Cl^−^ in vesicular trafficking and to confirm whether Cl^−^ homeostasis regulates the plant immunity response.

The recent identification of transporters involved in cell Cl^−^ influx will give a decisive boost to a better understanding of Cl^−^ nutrition. For this reason, it will be very important to identify which residues determine Cl^−^ selectivity in the substrate-binding pocket of NPF proteins. Natural variability of the Cl^−^ inclusion/exclusion rates exhibited by different plant species and varieties suggests the occurrence of an array of genes and alleles responsible for different NO_3_^−^ versus Cl^−^ selectivities (as observed for different members of the NPF and CLC families). Differential NO_3_^−^/Cl^−^ selectivities are expected to occur at different levels: The root–soil, the symplast–xylem, and the cytosol–vacuole interfaces. Characterization of Cl^−^ channels implicated in releasing Cl^−^ to the rhizosphere, which fine-tunes net Cl^−^ uptake in the root, is another issue that requires attention. Genes encoding these channels, as well as R-type channels involved in root-to-shoot Cl^−^ translocation and PM transport proteins involved in Cl^−^ allocation through the phloem, remain unexplored so far. The function of NPF transporters from the NAXT subfamily involved in Cl^−^ excretion from plant cells could be relevant under specific physiological conditions, possibly complementing those of S^−^ and R-type channels. However, this hypothesis requires further research.

New experiments are also required to better understand the localization and biological function of CCC transporters in order to clearly establish their biological functions. This and other endomembrane Cl^−^ transporters like AtALMT9, GmCLC1, GsCLC2, and possibly GmSALT3/CHX1 involved in intracellular Cl^−^ homeostasis have been proposed to regulate shoot Cl^−^ accumulation and salinity tolerance. In particular, intracellular Cl^−^ compartmentalization in vascular cells apparently plays a crucial, but still obscure, role in controlling whole-plant ion (e.g., Cl^−^ and Na^+^) distribution. Finally, more research on hormonal regulation and signal transduction processes that control Cl^−^ nutrition at the whole-plant level is urgently required.

## Figures and Tables

**Table 1:** Leaf Cl^−^ Concentration in different plant species grown with nutrient solutions containing Cl^−^ in the low milli-molar range (4.5–5.0 mM).

	Citrus Cl^−^ Excluder ^2^	*Arabidopsis* (Col0) ^3^	Citrus Cl^−^ Includer ^4^	Tomato ^5^	Tobacco ^6^
Leaf Cl^−^ Concentration (mg·g^−1^ DW)	5–13	25	30	32	50
Cl^−^ Excess (Respect to micronutrient requirement) ^1^	25–70	125	150	150	250

^1^ Considering 0.2 mg·g^−1^ DW as the critical Cl^−^ requirement. ^2^ Cleopatra mandarin and Rangpur lime plants (respectively) treated for 30 weeks with a nutrient solution containing 4.5 mM Cl^−^ [18]. ^3^ Arabidopsis thaliana (Columbia ecotype) treated for 5 weeks with a nutrient solution containing 5.0 mM Cl^−^ [20]. ^4^ Carrizo citrange plants treated for 30 weeks with a nutrient solution containing 4.5 mM Cl^−^ [18]. ^5^ Tomato plants treated for 6 weeks with a nutrient solution containing 5 mM Cl^−^ [19]. ^6^ Tobacco plants treated for 6 weeks with a nutrient solution containing 5 mM Cl^−^ [2].

**Table 2:** Major breakthrough regarding Cl^−^ function research in plants.

Year	Cl^−^ Function	Reference
1946	- Cl^−^ is required for **photochemical activity** in washed chloroplasts - Cl^−^ is proposed to be an essential micronutrient	[123]
1954	- Demonstration of Cl^−^ as **essential micronutrient** in tomato plants	[1]
1956	- Demonstration of Cl^−^ as **essential micronutrient** in other plant species	[17]
1963	- Cl^−^ is required for **Oxygen evolution in Photosystem-II**	[124]
1977	- The requirement of Cl^−^ is **not limited to photosynthesis** - Cl^−^ is also required for **adequate cell division** rate in the leaves	[47]
1980	- Cl^−^ regulates the **activity of some proteins**	[14,15]
1987	- Some plant species **require Cl^−^ beyond micronutrient levels**	[112,113,114]
1991	- Cl^−^ has a more **tightly bound hydration shell**	[58,59]
2009	- Localization and **role of Cl^−^ in oxygen-evolving Photosystem II**	[13]
2014	- Cl^−^ is required for adequate **cell elongation**	[2,51]
2015	- **Occurence of Cl^−^ deficiency in agricultural soils** is demonstrated for a relevant crop species	[112]
2016	- Cl^−^ specifically improves **osmoregulation, water balance and turgor** - Cl^−^ is proposed as a **beneficial macronutrient**	[2]
2016	- Chloroplast Cl^−^ homeostasis **regulates photosynthetic electron transport and photoprotective mechanisms**	[97,98]
2019	- As beneficial macronutrient Cl^−^ **improves water-use efficiency** by reduced stomatal conductance and increased mesophyll diffusion to CO_2_	[23]

**Table 3:** Functional properties of Cl^−^ transport proteins potentially involved in Cl^−^ nutrition: net uptake and/or long-distance transport.

Protein Name	Localization	Cell Function	Biological Role	References
ZmNPF6.4	Plasma membrane of root and shoot tissues	High affinity Cl^−^ selective H^+^/Cl^−^ symporter Cl^−^ influx	Undetermined	[147]
ZmALMT1	Plasma membrane of mature root tissues	R-type NO_3_^−^ > sulfate > Cl^−^ selective anion channel Anion efflux	Undetermined	[167]
AtNPF2.5	Plasma membrane of root cortical cells	Low magnitude Cl^−^ cell efflux	Cl^−^ exclusion through excretion to the rhizosphere Salinity tolerance?	[153]
AtSLAH3	Plasma membrane of guard cells Plasma membrane of Xylem-pole pericycle cells	S-type NO_3_^−^ > Cl^−^ selective anion channel High magnitude NO_3_^−^ and Cl^−^ cell efflux	Stomatal closure Root xylem loading of NO_3_^−^ and Cl^−^	[45,176]
AtSLAH1	Plasma membrane of Xylem-pole pericycle cells	Electrically silent S-type anion channel Regulation of SLAH3 activity	Regulation of root-to-shoot Cl− conductance	[176,184]
AtNPF2.4	Plasma membrane of root stelar cells	Low magnitude Cl^−^ cell efflux	Root-to-shoot Cl^−^ translocation	[152]
AtALMT12	Plasma membrane of guard cells Root vasculature	R-type malate-activated NO_3_^−^ > Cl^−^ selective anion channel Outward NO_3_^−^ and Cl^−^ cell efflux	Stomatal closure Undetermined root function	[185,186]
AtCCC	Golgi and trans-Golgi network Plasma membrane? Root vasculature and many other tissues	Cl^−^-dependent Na^+^/K^+^ Cotransporter	Directly or indirectly affects root-to-shoot Cl− distribution Developmental and other undetermined functions	[56,122]
OsCCC1	Plasma membrane of multiple root and shoot cell types	Cl^−^-dependent Na^+^/K^+^ Cotransporter	Regulation of Cl^−^, Na^+^ and K^+^ homeostasis and cell osmotic potential	[57]
AtALMT9	Tonoplast of guard cells Root and shoot vasculature	R-type malate-activated vacuolar Cl^−^ channel Cl^−^ homeostasis during early salinity stress	Required for stomatal opening Regulation of ion xylem loading	[69,70]
AtCLCc	Tonoplast of root, pollen grain and guard cells	vacuolar Cl^−^ compartmentalization	Stomatal function Regulations of shoot Cl^−^ accumulation? Salinity tolerance	[198,199]
AtCLCg	Tonoplast of mesophyll cells vascular tissue	vacuolar Cl^−^ compartmentalization	Regulations of shoot Cl^−^ accumulation? Salinity tolerance	[201]
GmCLC1	Tonoplast	vacuolar pH-dependent Cl^−^ compartmentalization H^+^/Cl^−^ Antiporter	Root Cl^−^ sequestering Regulations of shoot Cl^−^ accumulation Salinity tolerance	[194,195,196]
GsCLC-c2	Tonoplast	vacuolar channel with Cl^−^ > NO_3_^−^ affinity Cl^−^ and NO_3_^−^ compartmentalization	Regulations of shoot Cl^−^ accumulation? Salinity tolerance	[200]
GmSALT3/CHX1	Endoplasmic reticulum of root vasculature-associated cells	Unknown	Regulations of shoot Cl^−^ accumulation? Salinity tolerance	[210,211,212]
